# High-Recall Biomedical Language Models for Radiation Oncology Evidence Synthesis: An Integrated Systematic Review of Prediction Models for Radiotherapy-Induced Complications in Nasopharyngeal Carcinoma

**DOI:** 10.3390/life16091566

**Published:** 2026-09-18

**Authors:** Guang-Zhi Lin, Yang-Wei Hsieh, Chih-Yu Cheng, Chen-Cheng Wang, Liyun Chang, Chao-Hong Liu, Wen-Ping Yun, Shyh-An Yeh, Cheng-Shie Wuu, Chien-Liang Chiu, Pei-Ju Chao, Tsair-Fwu Lee

**Affiliations:** 1 Medical Physics and Informatics Laboratory of Electronic Engineering, National Kaohsiung University of Science and Technology, Kaohsiung 80778, Taiwan; haguy0507@gmail.com (G.-Z.L.); wewe750422@gmail.com (Y.-W.H.); f114152170@nkust.edu.tw (C.-Y.C.); 1106405106@nkust.edu.tw (W.-P.Y.);; 2 Department of Radiation Oncology, Kaohsiung Veterans General Hospital, Kaohsiung 813414, Taiwan; 3 Department of Medical Imaging and Radiological Sciences, I-Shou University, Kaohsiung 82445, Taiwan; 4 Department of Dermatology, Kaohsiung Yuan’s General Hospital, Kaohsiung 80249, Taiwan; y4509@yuanhosp.com.tw; 5 Department of Radiation Oncology, E-DA Hospital, Kaohsiung 82445, Taiwan; 6 Department of Radiation Oncology, Columbia University, New York, NY 10032, USA; csw6@columbia.edu; 7 Department of Radiation Oncology, Kaohsiung Chang Gung Memorial Hospital and Chang Gung University College of Medicine, Kaohsiung 833401, Taiwan; 8 Graduate Institute of Clinical Medicine, Kaohsiung Medical University, Kaohsiung 807378, Taiwan; 9 Department of Medical Imaging and Radiological Sciences, Kaohsiung Medical University, Kaohsiung 80708, Taiwan

**Keywords:** systematic review, nasopharyngeal carcinoma, radiotherapy complications, pretrained language models, abstract screening

## Abstract

Background: Radiotherapy-related complications can impair long-term outcomes in nasopharyngeal carcinoma (NPC), while high-volume literature screening remains a bottleneck in evidence synthesis. Methods: Four databases were searched through January 2026. After deduplication, 4916 records underwent two-stage manual screening, yielding 38 studies. Three Bidirectional Encoder Representations from Transformers (BERT) models, BERT-base, BioBERT, and PubMedBERT, were fine-tuned using stratified five-fold cross-validation, and screening performance was assessed using the area under the receiver operating characteristic curve (ROC-AUC), the area under the precision-recall curve (PR-AUC), and work saved over sampling (WSS) at the 100% and 85% recall thresholds (WSS@100% and WSS@85%).The clinical evidence was evaluated using the Prediction Model Risk of Bias Assessment Tool (PROBAST) and synthesized narratively according to endpoint, discrimination metric, time horizon, unit of analysis, validation level, and uncertainty reporting. Results: At complete recall, BioBERT and PubMedBERT achieved mean WSS values of 97.8% and 97.5%, respectively, compared with 89.7% for BERT-base. Separately, the clinical evidence review identified 39 discrimination estimates: 33 conventional binary ROC-AUCs, three Harrell’s C-indices, and three time-dependent AUCs. Only two studies contributed external-validation estimates and one contributed a temporal-validation estimate. Sixteen estimates lacked a usable measure of uncertainty, and PROBAST rated all studies as having a high overall risk of bias, driven by the analysis domain. Conclusions: Within the retrieved and manually annotated corpus, biomedical pretrained language models showed the potential to reduce the title-and-abstract screening burden while maintaining complete recall. Independently, the clinical evidence map identified promising discrimination but insufficiently robust and transportable evidence for routine clinical implementation.

## 1. Introduction

As the volume of medical literature continues to grow, systematic reviews remain essential to evidence-based medicine. However, manually screening thousands or tens of thousands of bibliographic records remains labor-intensive and time-consuming. Because relevant studies often account for less than 3% of retrieved records, title-and-abstract screening can consume substantial reviewer time and delay full-text assessment, data extraction, risk-of-bias evaluation, and meta-analysis [1].

Artificial intelligence (AI), particularly natural language processing (NLP), has emerged as a potential approach to streamline literature-screening workflows. Early deep learning applications in document classification often used recurrent neural network architectures, including long short-term memory and gated recurrent units [2]. However, these sequential architectures may have difficulty capturing long-range semantic dependencies and specialized biomedical terminology. Recent research has increasingly adopted Transformer models with attention mechanisms. Biomedical pretrained language models such as BioBERT and PubMedBERT may better represent clinical terminology and complex semantic relationships than generic sequence models [3,4,5].

Nasopharyngeal carcinoma (NPC) radiotherapy represents one clinical setting in which rigorous evidence synthesis is needed. Treatment-related complications (including xerostomia, severe oral mucositis, hypothyroidism, temporal lobe injury, and radiation dermatitis) span acute and late toxicities that can impair long-term quality of life and influence follow-up care [6,7]. Clinical, radiomic, dosimetric, and machine learning models have been developed to predict these outcomes, but they differ substantially in target endpoints, model architectures, validation strategies, and reporting quality. A systematic review is therefore needed to characterize the reported discrimination measures, validation practices, methodological limitations, and clinical applicability of these models.

Several recent systematic reviews have addressed clinically related aspects of post-radiotherapy complication prediction in head-and-neck cancer. Takada et al. reviewed prognostic and prognostic and normal tissue complication probability (NTCP) models across head-and-neck cancer populations and identified limited external validation, frequent high risk of bias, and insufficient evidence for clinical implementation [8]. Dong et al. conducted an endpoint-specific meta-analysis of dosimetric predictors for radiation-induced temporal lobe necrosis in NPC [9], while Tajiki et al. summarized NTCP models and radiobiological parameters across head-and-neck cancer endpoints [10]. Together, these reviews provide important toxicity-specific and radiobiological context. The present review complements them by focusing specifically on the methodological characteristics, validation practices, risk of bias, and clinical applicability of multi-endpoint prediction models for radiotherapy-related complications in NPC.

Workload-saving metrics such as work saved over sampling (WSS) at 95% recall (WSS@95%) are increasingly used to evaluate automated or semi-automated screening tools [11]. However, most methodological studies focus on classification performance or workload reduction during title-and-abstract screening [12], and few examine how screening assistance fits within a complete evidence-synthesis workflow [13]. The practical value of NLP-assisted screening should therefore be evaluated under high-recall conditions and interpreted in terms of the potential reduction in records requiring manual review. Such reductions may allow more reviewer effort to be directed toward full-text assessment and evidence synthesis, although this downstream benefit requires prospective workflow evaluation.

A previous study by our group demonstrated the feasibility of integrating a supervised Bidirectional Encoder Representations from Transformers (BERT) classifier with a meta-analytic workflow for evidence synthesis on radiotherapy-induced complications in NPC [14]. The present study was conducted as a separate review and did not continue, update, or reuse the literature corpus, screening records, or article-level dataset of the earlier Reports article. However, because the earlier publication did not report a study-level list of included articles and its original article-level screening dataset was not available for reliable reconstruction, incidental overlap between individual included publications could not be independently verified. The present study addresses a distinct methodological and clinical synthesis question and is differentiated from the earlier work by its documented research question, newly conducted search and screening process, comparison of three pretrained language models, stratified five-fold cross-validation, recall-focused workload metrics, and expanded clinical evidence-synthesis framework.

Accordingly, this study had two objectives: to compare BERT-base, BioBERT, and PubMedBERT for high-recall title-and-abstract screening within the manually annotated corpus, and to systematically characterize prediction models for radiotherapy-induced complications in NPC according to clinical endpoint, discrimination metric, time horizon, validation level, uncertainty reporting, risk of bias, and applicability.

## 2. Methods

### 2.1. Study Framework

**Figure 1:**
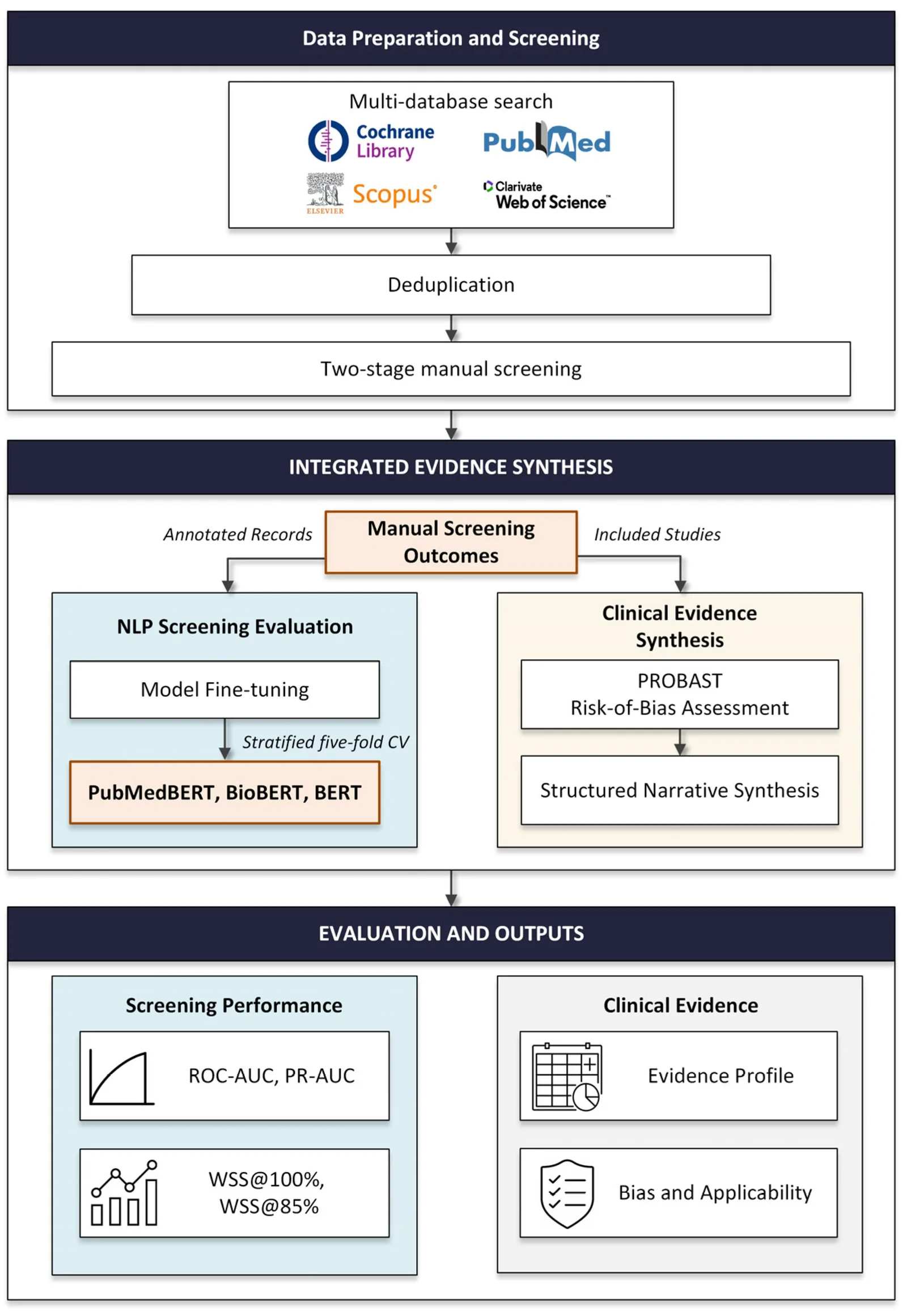
Integrated workflow for NLP screening evaluation and clinical evidence synthesis. Records retrieved from PubMed, Web of Science, Scopus, and the Cochrane Library were deduplicated and underwent two-stage manual screening. The resulting annotated records were used to fine-tune and evaluate PubMedBERT, BioBERT, and BERT using stratified five-fold cross-validation. Screening performance was assessed using ROC-AUC, PR-AUC, WSS@100%, and WSS@85%. Included studies underwent PROBAST risk-of-bias assessment and structured narrative synthesis to characterize the clinical evidence profile, risk of bias, and applicability. No cross-study pooling of clinical performance estimates was performed. **Abbreviations:** BERT, Bidirectional Encoder Representations from Transformers; BioBERT, Biomedical Bidirectional Encoder Representations from Transformers; CV, cross-validation; NLP, natural language processing; PR-AUC, area under the precision–recall curve; PROBAST, Prediction Model Risk of Bias Assessment Tool; PubMedBERT, PubMed-pretrained Bidirectional Encoder Representations from Transformers; ROC-AUC, area under the receiver operating characteristic curve; WSS, Work Saved over Sampling. WSS@100% and WSS@85% denote WSS evaluated at recall thresholds of 100% and 85%, respectively.

This study used an integrated dual-track framework that linked NLP screening evaluation with structured clinical evidence synthesis. As illustrated in Figure 1, the workflow comprised data preparation and screening, integrated evidence synthesis, and evaluation and outputs. This systematic review and meta-analysis was reported in accordance with the Preferred Reporting Items for Systematic Reviews and Meta-Analyses 2020 statement (PRISMA 2020).

Records retrieved from PubMed, Web of Science, Scopus, and the Cochrane Library were deduplicated and underwent two-stage manual screening. The manual screening outcomes generated an annotated record-level corpus for NLP evaluation and a set of included full-text studies for clinical evidence synthesis.

The NLP track compared BERT-base, BioBERT, and PubMedBERT using stratified five-fold cross-validation. The clinical track comprised structured data extraction, classification of discrimination metrics and validation settings, Prediction Model Risk of Bias Assessment Tool (PROBAST) risk-of-bias and applicability assessment, and endpoint-specific narrative synthesis. Because the NLP models were applied only after database retrieval and deduplication, they ranked records within the retrieved corpus and could not identify studies absent from it.

NLP screening performance was evaluated using the area under the receiver operating characteristic curve (ROC-AUC), the area under the precision-recall curve (PR-AUC), WSS@100%, and WSS@85%. Clinical outputs comprised an evidence profile organized by endpoint, metric family, time horizon, unit of analysis, validation level, uncertainty reporting, risk of bias, and applicability. No cross-study pooling of clinical performance estimates was performed.

### 2.2. Data Preparation

#### 2.2.1. Search Strategy and Multi-Database Search

The search strategy was developed according to the population, intervention, comparator, outcome, and study design (PICOS) framework. The population comprised patients with nasopharyngeal carcinoma receiving modern photon intensity-modulated radiotherapy techniques, namely intensity-modulated radiotherapy (IMRT), volumetric modulated arc therapy (VMAT), or TomoTherapy; the outcome of interest was the discriminative performance of prediction models quantified by the area under the curve (AUC); no restrictions were placed on comparators, and only original research articles were eligible by study design. Guided by this framework, a systematic search was conducted in PubMed, Web of Science, Scopus, and the Cochrane Library, with key concepts linked using Boolean operators (AND/OR). The search was restricted to English-language publications and covered the period from database inception to January 2026. The final searches of PubMed, Web of Science, Scopus, and the Cochrane Library were conducted on 22 January 2026. The outcome search block explicitly included hypothyroidism, temporal lobe injury or necrosis, lymphopenia, hearing loss, and hypoglossal neuropathy across all four databases. The complete database-specific search strings are provided in Supplementary Material S1. This systematic review was retrospectively registered in INPLASY on 8 June 2026 (registration number: INPLASY202660043; DOI: 10.37766/inplasy2026.6.0043).

#### 2.2.2. Eligibility Criteria

Studies were included if they met all of the following criteria: (1) development or validation of prediction models for radiation-induced complications in nasopharyngeal carcinoma; (2) reporting AUC as the measure of discriminative performance; and (3) publication as an original research article in a peer-reviewed journal.

Studies were excluded if they met any of the following criteria: (1) use of non-modern photon intensity-modulated radiotherapy techniques, including three-dimensional conformal radiotherapy (3D-CRT) or particle therapy; (2) failure to report AUC as a measure of model discriminative performance; or (3) non-original research, including reviews, editorials, conference abstracts, and case reports.

The AUC-based eligibility criterion was retained as originally applied during screening. For included time-to-event studies that reported both an AUC-based measure and a C-index, the primary discrimination measure designated by the original authors was retained for interpretation. This post hoc metric audit did not retrospectively alter record-level eligibility labels or the NLP reference standard.

#### 2.2.3. Deduplication and Two-Stage Manual Screening

Search results from the four databases were imported into EndNote 20 for reference management and deduplication. Two reviewers then independently conducted two-stage screening. Titles and abstracts were screened first to exclude clearly irrelevant records. Full texts of potentially eligible records were subsequently assessed against all inclusion criteria, and reasons for exclusion were documented (Supplementary Table S5). Disagreements were resolved through discussion; when consensus could not be reached, a third reviewer adjudicated.

#### 2.2.4. Screening Results and Data Annotation

The results of manual screening served as the reference standard for subsequent fine-tuning and evaluation of the NLP models, forming the basis of the NLP screening dataset. Studies that were finally included in the systematic review were annotated as relevant, whereas all other non-included records were annotated as non-relevant.

### 2.3. NLP Models

#### 2.3.1. Model Selection

This study compared three BERT-family models: a general-domain pretrained model (BERT-base) and two biomedical domain-specific pretrained models (BioBERT and PubMedBERT). BERT-base, trained primarily on general corpora such as Wikipedia, served as the baseline. BioBERT was obtained through domain-adaptive pretraining on PubMed corpora from general BERT weights, thereby incorporating both general linguistic and biomedical information. PubMedBERT was pretrained entirely on biomedical literature using a domain-specific vocabulary designed to better represent biomedical terminology.

#### 2.3.2. Model Fine-Tuning and Stratified Five-Fold Cross-Validation

For each record, the title and abstract were concatenated into a single input sequence and encoded using the tokenizer corresponding to each pretrained model. The same fine-tuning settings were applied across all three models. Stratified sampling maintained a similar proportion of relevant studies in each fold, reducing the risk that positive records would be concentrated in or absent from a fold. In stratified five-fold cross-validation, the training folds were used for fine-tuning and the corresponding held-out fold was used to evaluate high-recall screening performance. All fold-dependent preprocessing, fine-tuning, validation, hyperparameter tuning, and checkpoint selection were performed without access to the held-out test fold. Each record served as test data exactly once, maximizing use of the available annotated corpus, while fold-level means and standard deviations provided an objective estimate of internal screening performance and its variability across the five splits. Because all folds originated from the same retrieved and manually annotated corpus, this evaluation remains internal and does not constitute prospective or external validation.

For each outer cross-validation split, four folds formed the training pool and the remaining fold served as the held-out test set. A stratified 10% validation subset was further separated from the training pool. The held-out test fold was not used for hyperparameter tuning or checkpoint selection. The specific pretrained checkpoints were bert-base-uncased [15], dmis-lab/biobert-base-cased-v1.1 [4], and microsoft/BiomedNLP-PubMedBERT-base-uncased-abstract [5]. The same fold assignments, random seed (42), and hyperparameter settings were applied to all three models. Each model was fine-tuned for 12 epochs using a maximum sequence length of 256, a batch size of 4, dropout of 0.3, standard cross-entropy loss without class weighting, and the AdamW optimizer with a one-epoch linear warm-up followed by cosine annealing. The checkpoint with the highest validation PR-AUC was retained; ties were resolved first by selecting the checkpoint with the lower validation loss and then, if necessary, the earlier epoch. No chemicals, reagents, commercial cell lines, or physical measurement instruments were used in this study. All analyses were performed in Python 3.10.19 with PyTorch 2.7.1+cu118, Transformers 5.0.0, scikit-learn 1.7.2, NumPy 2.2.6, pandas 2.3.3, and SciPy 1.15.2.

#### 2.3.3. Evaluation Metrics and Definition of Operating Points

ROC-AUC and PR-AUC were calculated directly from the continuous predicted relevance probabilities. Within each held-out test fold, records were ranked in descending order of predicted relevance. Let N denote the total number of records in the test fold, n_screened the minimum number of top-ranked records that had to be screened to achieve the target recall, and R the recall actually achieved. Work Saved over Sampling was calculated using the conventional definition [11]: WSS@R = R − n_screened/N. At the 100% recall operating point, this simplifies to WSS@100% = 1 − n_screened/N. The nominal 85% recall operating point was defined as the minimum ranking position required to achieve at least 85% recall. Because the number of relevant records in each fold was discrete, the mean recall actually achieved at this operating point was 0.868 ± 0.010. No fixed probability threshold was used to calculate ROC-AUC, PR-AUC, or WSS, and these rank-based operating points should not be interpreted as fixed thresholds for prospective deployment.

### 2.4. Clinical Evidence Synthesis

#### 2.4.1. Data Extraction and Evidence Classification

Two reviewers independently extracted study characteristics, predicted complications, model types, predictor domains, discrimination measures, time horizons, units of analysis, evaluation cohorts, cohort sizes, event and non-event counts when available, validation procedures, and reported uncertainty. When a study reported multiple candidate models, the final or primary model designated by the original authors was selected to reduce double counting and selective inclusion.

For time-to-event studies, the primary discrimination measure specified by the original authors was retained rather than replaced by a secondary time-specific estimate. Reported measures were classified as conventional binary ROC-AUC, Harrell’s C-index, or time-dependent AUC. Validation was classified as development or training only, held-out internal validation, bootstrap or other internal validation, cross-validation, external validation, or temporal validation. The sample size recorded for each estimate was the cohort that actually generated that estimate rather than the total study population.

For the four major toxicity groups, data extraction additionally covered the primary organ at risk or anatomical structure; clinical, dosimetric, treatment-related, imaging, radiomic, and omics predictors; and validation-related limitations. Predictor summaries distinguished variables evaluated as candidates from those retained in final models or reported recurrently across studies.

#### 2.4.2. Risk-of-Bias and Applicability Assessment

Two reviewers independently assessed the methodological quality of the included prediction-model studies using PROBAST. Disagreements were resolved through discussion; when consensus could not be reached, a third reviewer adjudicated. Risk of bias was evaluated across the participants, predictors, outcome, and analysis domains, whereas applicability was evaluated across the participants, predictors, and outcome domains. Domain-level judgments were categorized as having low, high, or unclear risk of bias or concern, and the overall judgment followed PROBAST guidance.

#### 2.4.3. Synthesis Approach

Clinical evidence was synthesized narratively and organized by clinical endpoint, discrimination metric, time horizon, unit of analysis, validation level, evaluation cohort, and availability of uncertainty information. Estimates based on different metric families or time horizons were not treated as a common effect measure. Confidence intervals retained in the evidence tables were transcribed as reported in the original publications and were not recalculated. Standard errors were not reconstructed from the reported AUC and total study sample size, and a total study sample size was not substituted when the size of the actual evaluation cohort was unavailable. Study-level estimates were retained in the evidence tables to preserve their original analytical context, and no cross-study pooling of clinical performance estimates was performed.

Meta-regression, funnel-plot asymmetry testing, trim-and-fill procedures, prediction intervals, and leave-one-out meta-analytic sensitivity analyses were not performed because a common effect measure with valid study-level variances was unavailable [16,17].

Following retrospective registration, the planned meta-analysis was replaced with a structured narrative synthesis because heterogeneity in clinical endpoints, discrimination metrics, time horizons, units of analysis, validation settings, and uncertainty reporting precluded defensible statistical pooling. The search strategy, eligibility criteria, screening process, and included-study set were unchanged. No formal Grading of Recommendations, Assessment, Development, and Evaluations (GRADE) certainty-of-evidence assessment was conducted.

## 3. Results

### 3.1. Study Selection and Construction of the NLP Dataset

**Figure 2:**
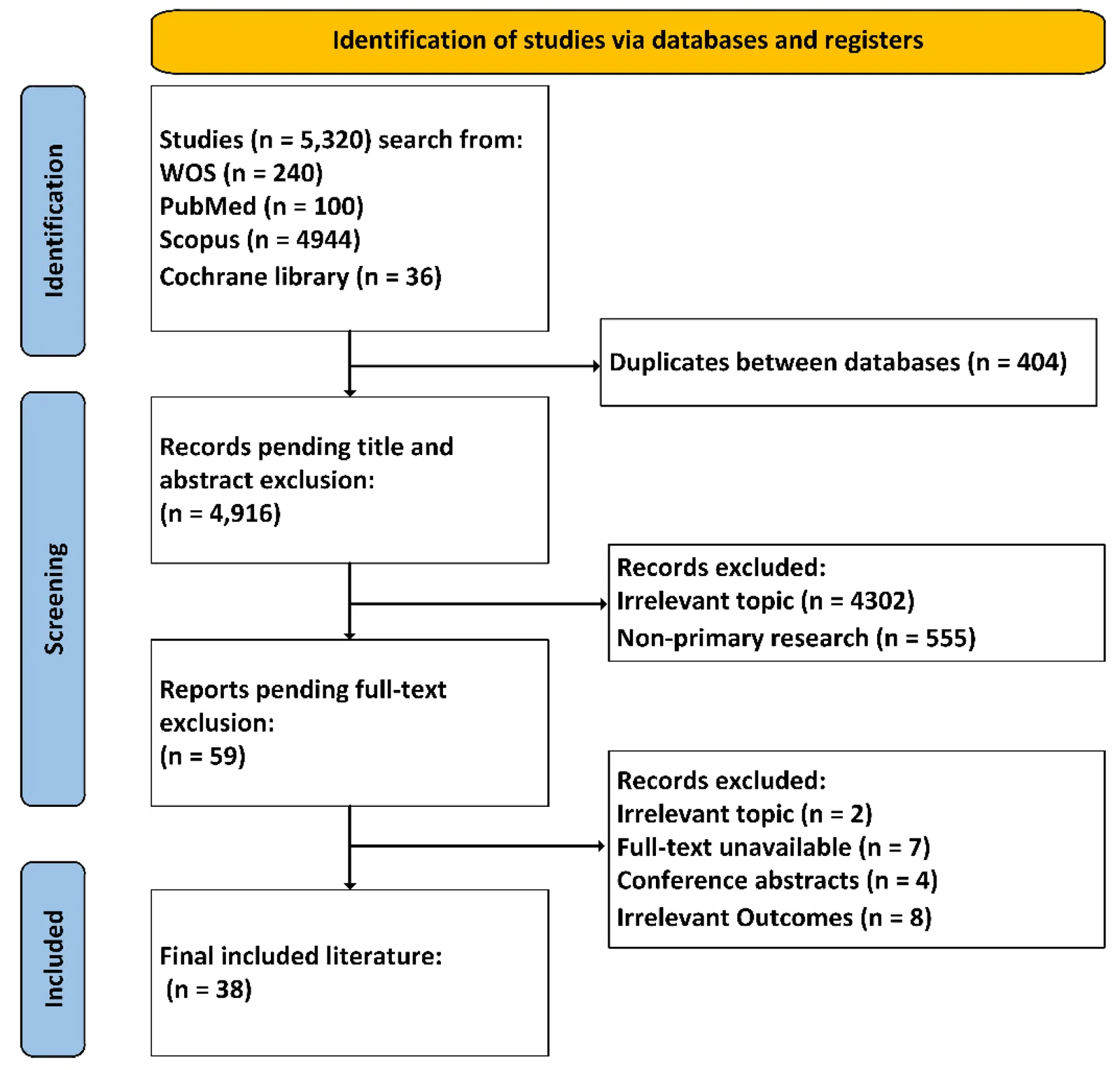
Literature selection flow diagram. The flow diagram summarizes database searching, deduplication, title-and-abstract screening, full-text assessment, exclusion reasons, and final inclusion of studies in the systematic review. **Abbreviations:** *n*, number of records; WOS, Web of Science.

A total of 5320 records were retrieved from the four databases, of which 4916 remained after deduplication and proceeded to the screening process. Following a two-stage screening of titles/abstracts and full texts, 38 studies that met the eligibility criteria were included. The detailed study selection process is presented in Figure 2.

### 3.2. Characteristics of Included Studies

All 38 included studies evaluated models for predicting radiation-induced complications in NPC. Publication years ranged from 2014 to 2026, and sample sizes ranged from 78 to 8194. Most studies were conducted in China (*n* = 33). The remaining studies were conducted in Taiwan, Italy, or Thailand, or were multicenter studies. Reported complications included temporal lobe injury or necrosis (TLI/RTLI/TLN), severe oral mucositis (SOM), xerostomia, hypothyroidism, radiation dermatitis, grade 4 lymphopenia, radiation-induced hypoglossal neuropathy, hearing loss, middle-ear effusion, nasal-cavity stenosis, and other late radiation-related injuries.

The prediction models encompassed traditional statistical models (such as logistic regression and Cox regression), regularized methods such as the least absolute shrinkage and selection operator (LASSO), NTCP models, and machine learning models such as support vector machines, random forests, extreme gradient boosting (XGBoost), and ensemble methods. Detailed characteristics of the included studies are summarized in Table 1 [18,19,20,21,22,23,24,25,26,27,28,29,30,31,32,33,34,35,36,37,38,39,40,41,42,43,44,45,46,47,48,49,50,51,52,53,54,55].

To provide a clinically oriented synthesis of the included evidence, the four major toxicity groups were additionally summarized according to their primary organs at risk, recurrent dosimetric or clinical predictor patterns, and validation-related limitations (Table 2).

The four major endpoint groups showed distinct clinical and predictor profiles. Models of cerebral radiation injury or necrosis primarily focused on temporal-lobe dosimetry and imaging-derived features, whereas severe oral mucositis models incorporated oral-cavity or mucosal dose exposure together with clinical and treatment-related variables. Xerostomia models were predominantly related to parotid-gland exposure, while hypothyroidism models emphasized thyroid dose-volume parameters and selected clinical characteristics. These differences support endpoint-specific interpretation rather than treating the outcomes as interchangeable manifestations of a single generic radiotherapy complication. Most models remained dependent on internal validation, and independent external validation was limited.

### 3.3. Clinical Evidence Profile

#### 3.3.1. Metric, Validation, and Uncertainty Profile

The 38 included prediction-model studies contributed 39 study-level discrimination estimates because Wen et al. reported both a primary C-index and a secondary 5-year time-dependent AUC. Of the 39 estimates, 33 were conventional binary ROC-AUCs, three were time-to-event Harrell’s C-indices, and three were time-dependent AUCs. The time-dependent estimates were reported at horizons ranging from one month to five years, and the units of analysis included both patients and temporal lobes.

Two studies contributed an external-validation estimate and one contributed a temporal-validation estimate. The remaining estimates comprised 25 held-out internal validations, five bootstrap or other internal validations, two cross-validation estimates, and three development- or training-cohort estimates. In four studies, the size of the cohort that actually produced the selected performance estimate was not reported; the total study population was therefore not used as a substitute.

No selected estimate was accompanied by a directly reported standard error or variance. Twenty-one estimates included a reported 95% confidence interval, two provided complete event and non-event counts for the actual evaluation cohort, ten were reported only as point estimates, and six had unclear metric definitions, time horizons, evaluation cohorts, or numerical provenance. These categories indicate that the uncertainty and analytical context required for reliable cross-study weighting were unavailable for a substantial proportion of the evidence base.

#### 3.3.2. Endpoint-Specific Evidence

The principal evidence groups were cerebral radiation injury or necrosis (11 studies), severe oral mucositis (nine studies), xerostomia occurrence or severity (five studies), and radiation-induced hypothyroidism (four studies). The remaining nine studies addressed xerostomia recovery, a composite central nervous system endpoint, radiation dermatitis, grade 4 lymphopenia, hypoglossal neuropathy, sensorineural hearing loss, acquired nasal cavity stenosis and atresia, or composite late radiation injuries. Study-level metrics, evaluation cohorts, validation levels, and uncertainty information are reported in Table 1 and Supplementary Table S4.

Cerebral injury models most commonly incorporated temporal-lobe dose measures, clinical factors, or radiomic features. Oral mucositis models used combinations of clinical, treatment-related, dosimetric, oral-health, imaging, or multi-omic predictors. Xerostomia models focused on salivary-gland dose or imaging features, whereas hypothyroidism models emphasized thyroid dose-volume measures and clinical characteristics. Although favorable discrimination was frequently reported, most estimates were derived from internal validation, and the clinical meaning of the reported values depended on the endpoint definition, metric family, time horizon, and validation setting.

For Wen et al. (2021) [25], the primary performance measure designated by the authors was Harrell’s C-index of 0.775 (95% CI 0.751–0.799) in a test set of 4916 temporal lobes. The reported 5-year AUC of 0.818 was a secondary time-dependent estimate from the same test set and was not accompanied by a confidence interval or by event and censoring information specific to the 5-year horizon. The two measures were therefore retained as distinct estimands and were not used interchangeably.

Taken together, the evidence base was distributed across clinical endpoints, metric families, time horizons, validation settings, and units of analysis, with most combinations represented by only one or a few studies. Incomplete uncertainty information further limited reliable cross-study weighting. The results were therefore organized as a structured evidence profile rather than reduced to a pooled estimate, allowing findings with independent validation to be distinguished from those supported only by development or single-cohort evaluations.

### 3.4. Methodological Quality, Risk of Bias, and Applicability

PROBAST rated all 38 studies as having a high overall risk of bias because the analysis domain was judged to be at high risk in every study. In contrast, the participant, predictor, and outcome domains were rated as low risk in 32, 37, and 36 studies, respectively. The most frequently identifiable analysis-domain concerns were predictor selection based on univariable analyses (20 studies), absence of calibration assessment (14 studies), and clearly inadequate sample sizes or event counts relative to model complexity (12 studies); a further 13 studies raised possible sample-size concerns. Additional concerns included insufficient control of overfitting or optimism, inappropriate handling of censoring or clustered observations, and incomplete reporting of missing-data and validation procedures. Confirmed evidence of data leakage was limited to one study; for several others, the reporting was insufficient to establish whether preprocessing, feature selection, and validation had been conducted independently. Because no study was classified as having a low overall risk of bias, a sensitivity analysis restricted to low-risk studies was not feasible.

Applicability concerns were less widespread than risks of bias. Overall applicability was rated as low concern in 34 studies and high concern in four, with the latter judgments arising from the participant domain. Predictor and outcome applicability were rated as low concern in all 38 studies. Thus, although most models addressed clinically relevant populations, predictors, and outcomes, limitations in their development and evaluation reduced confidence in the reported performance estimates. Detailed results are provided in Supplementary Table S1.

### 3.5. Screening Performance of Pretrained Language Models

Separately from the clinical evidence synthesis, the three pretrained encoders were evaluated as prioritization tools for title-and-abstract screening; they were not components of the clinical prediction models reported in the 38 included studies. Their performance is summarized in Table 3. PubMedBERT and BioBERT achieved comparable mean ROC-AUC values of 0.995 ± 0.005 and 0.995 ± 0.004, respectively, compared with 0.983 ± 0.020 for BERT-base. BioBERT had the highest mean PR-AUC at 0.706 ± 0.200, followed by BERT-base at 0.664 ± 0.175 and PubMedBERT at 0.647 ± 0.161. At the high-recall operating points relevant to screening prioritization, both domain-specific encoders yielded higher mean WSS@100% than BERT-base in the held-out folds. These descriptive within-corpus results indicate potential workload reduction at complete recall. Because simpler baseline methods were not evaluated and between-model differences were not formally tested, they do not establish superiority of the domain-specific encoders.

At the 100% recall operating point, BioBERT and PubMedBERT achieved mean WSS values of 97.8% ± 1.1% and 97.5% ± 2.0%, respectively, compared with 89.7% ± 11.9% for BERT-base. The corresponding potential screening burdens were approximately 2.2–2.5% of the test-fold records at complete recall. BERT-base showed substantially greater cross-fold variability at this operating point. At the nominal 85% recall operating point, at which the mean recall actually achieved was 0.868 ± 0.010, PubMedBERT and BioBERT produced comparable WSS values of 85.3% ± 0.9% and 85.2% ± 1.1%, respectively, while BERT-base achieved 83.3% ± 3.5%. These results indicate potential workload reduction in an offline ranking evaluation and should not be interpreted as prospectively observed savings in reviewer time.

Other screening-related performance metrics at the predefined operating points, including precision, specificity, and negative predictive value (NPV), are provided in Supplementary Tables S2 and S3.

## 4. Discussion

This review addressed two questions that are usually handled separately: whether biomedical pretrained language models can reduce the title-and-abstract screening burden without missing eligible studies, and what the available prediction-model literature supports for radiotherapy-related complications in NPC. The 38 included studies used heterogeneous outcomes, discrimination measures, follow-up horizons, and validation settings, so most clinically comparable evidence groups were supported by only one or a few studies, and all were rated as having a high overall risk of bias driven by the analysis domain. The findings are therefore presented as a structured evidence profile rather than as a single summary performance value.

### 4.1. Study Context

The present study is methodologically related to, but distinct from, our earlier BERT-assisted evidence-synthesis study [14]. The earlier work established the feasibility of integrating a supervised BERT classifier with a meta-analysis of post-radiotherapy complications in NPC. The present study was conducted as a separate review and did not continue, update, or reuse the earlier literature corpus, screening records, or article-level dataset. Because the earlier study-level dataset could not be reliably reconstructed, incidental overlap between individual included publications could not be independently verified. Instead, the present study evaluates a distinct methodological question by directly comparing BERT-base, BioBERT, and PubMedBERT under the same stratified five-fold cross-validation framework.

The evaluation emphasis also differs. Rather than focusing primarily on a single classifier, optimizer-related implementation choices, and execution time, the present study examines the incremental value of biomedical domain-specific pretraining using recall-focused measures of screening safety and potential workload reduction, particularly WSS at 100% recall. Its evidence-synthesis component uses the four-database search conducted for the present study, structured classification of discrimination metrics and validation settings, PROBAST-based appraisal, and endpoint-specific narrative synthesis.

### 4.2. Key Findings and Interpretation

Within the cross-validation test folds, all three BERT-family models achieved high WSS values at high-recall operating points using only titles and abstracts [56,57]. Because initial screening should minimize missed relevant studies while reducing manual review, WSS@100% recall was selected as the principal measure of potential workload reduction. At this operating point, BioBERT and PubMedBERT achieved mean WSS values of 97.8% and 97.5%, respectively, compared with 89.7% for BERT-base, and both biomedical models showed lower cross-fold variability. The descriptive pattern is consistent with a possible contribution of biomedical domain pretraining [5]. Its interpretation remains limited by the absence of simpler information-retrieval and conventional machine-learning baselines, the lack of formal between-model testing, and evaluation within a single review corpus.

The zero-miss performance was conditional on the retrieved and manually annotated corpus. The models ranked records only after database retrieval and therefore did not establish that the search captured every eligible study or replace manual eligibility assessment. The study also did not prospectively measure reviewer-time savings or improvements in downstream evidence quality.

### 4.3. Interpretation of the Clinical Evidence

The clinical evidence cannot be represented by a single summary discrimination value. Conventional binary ROC-AUC evaluates discrimination for a binary outcome definition, Harrell’s C-index evaluates concordance across observed time-to-event ordering under censoring, and time-dependent AUC evaluates discrimination at a specified horizon. Treating these measures as interchangeable would obscure differences in estimand, follow-up, censoring, and clinical interpretation.

The validation profile further limits interpretation. Only two studies contributed external-validation estimates and one contributed a temporal-validation estimate, whereas most estimates arose from held-out internal validation, resampling, cross-validation, or development cohorts. Internal estimates may be useful during model development, but they provide weaker evidence of transportability to new institutions, populations, treatment protocols, and outcome-assessment settings.

PROBAST classified all 38 studies as having a high overall risk of bias, driven uniformly by the analysis domain. The predominance of analysis-related bias indicates that the principal limitation was not uniformly poor conduct across all PROBAST domains, but the statistical pathways used to develop and evaluate the models. Predictor selection based on univariable significance, low information-to-complexity ratios, limited calibration assessment, incomplete control of overfitting or optimism, and inadequate handling or reporting of censoring, clustered observations, missing data, or validation pipelines can produce unstable predictor sets, underestimated uncertainty, and optimistic discrimination. Importantly, inadequate reporting could not always be distinguished from inappropriate analysis: only one study provided clear evidence of data leakage, whereas several others did not describe their analysis pipelines sufficiently to establish independence. The high-risk judgments therefore do not establish that all models were invalid or had every listed deficiency, but indicate that their reported performance was not sufficiently robust for routine clinical implementation. Most studies nevertheless had low concerns regarding applicability, indicating that their populations, predictors, and outcomes were generally relevant to the clinical scope of this review. However, clinical relevance does not ensure methodological reliability, and the uniformly high analysis-domain risk of bias limits confidence in the reported performance estimates.

Endpoint-specific interpretation remains clinically useful despite the absence of pooling. Cerebral injury, severe oral mucositis, xerostomia, and hypothyroidism involve different organs at risk, latency patterns, predictor structures, and intended clinical decisions. Organizing the evidence by endpoint, metric, time horizon, and validation level preserves these distinctions and identifies where independent validation, calibration assessment, and decision-curve or other clinical-utility analyses are most needed.

The study consequently makes two complementary contributions. First, it provides an NPC-specific map of prediction-model evidence and its methodological limitations, including clinical endpoints, discrimination metrics, time horizons, validation settings, risk of bias, and clinical applicability. Second, it evaluates three existing pretrained encoders as high-recall prioritization tools within the manually annotated review corpus. Development of a new language model was outside the scope of this study. These tools are intended to support expert judgment. They may allow reviewer effort to be redirected from manual screening of the large volume of titles and abstracts unlikely to meet the inclusion criteria toward full-text assessment and clinically meaningful evidence interpretation. NLP-assisted screening may support future systematic reviews and, when clinically and statistically comparable evidence is available, downstream meta-analysis; it cannot compensate for incompatible estimands or inadequate primary-study reporting.

### 4.4. Limitations

This study has several limitations. First, the NLP models were evaluated on a single clinical topic, and the number of relevant studies was limited. Even with stratified five-fold cross-validation, each test fold contained few positive records, which may make estimates of recall-based operating points, PR-AUC, and WSS sensitive to the score distribution of individual studies [58].

Second, the study used an offline evaluation rather than a prospective human–machine workflow [12]. Accordingly, the approximately 97% WSS represents the potential reduction in records requiring review under a complete-recall ranking scenario, not observed reviewer-time savings. Deployment in a new review corpus would require independent calibration and prospective validation of an operational stopping rule.

Third, this study did not include baseline methods such as keyword-based search, term frequency–inverse document frequency (TF–IDF), Okapi BM25, or traditional machine learning classifiers [59], and therefore cannot fully exclude the possibility that part of the model performance gain arises from relatively obvious lexical cues embedded in the topic or inclusion criteria. Future studies that incorporate these baseline approaches will help more clearly delineate the relative advantages of biomedical domain-specific pretrained models over general-domain models and traditional methods.

Fourth, the clinical evidence was limited by substantial variation across toxicity endpoints, discrimination metrics, time horizons, units of analysis, predictor sets, and validation strategies. No selected estimate was accompanied by a directly reported standard error or variance, and 16 of the 39 estimates provided no usable measure of uncertainty. Only two studies contributed an external-validation estimate and one contributed a temporal-validation estimate. All included studies had a high overall risk of bias. In addition, discrimination alone does not establish calibration, clinical utility, or net benefit; favorable study-level values should therefore not be interpreted as evidence that the models are ready for routine clinical use.

Fifth, the original screening criteria required reporting of an AUC-based discrimination measure. The metric audit retained the primary C-index for included time-to-event studies when it was designated by the original authors, but the review may not capture otherwise eligible prognostic-model studies that reported only non-AUC discrimination measures. This limitation should be considered when interpreting the completeness of the clinical evidence map.

Sixth, the review was registered retrospectively after the review process had commenced. Although the registration documents the review methods and subsequent analytical decisions, retrospective registration provides less protection against selective methodological changes than prospective protocol registration and should be considered a methodological limitation.

Finally, although the outcome search block explicitly covered the major complications represented in the included studies, the completeness of the evidence base remained dependent on database indexing, the search strategy, and manual eligibility assessment. Studies not retrieved by the database searches or incorrectly excluded during manual screening could not be recovered by the downstream NLP classifiers; such omissions could affect the completeness and external validity of both the systematic review and its AI-assisted screening evaluation.

### 4.5. Future Directions

Future research should extend this framework to systematic reviews of other clinical topics, incorporate traditional information-retrieval baselines, adopt active-learning strategies, and implement prospective human–machine interactive screening workflows. Evaluations should record actual screening time, reviewer workload, error types, and collaborative decision-making processes to determine whether WSS estimated from offline ranking translates into measurable savings in systematic review practice.

Beyond conventional ranking and active-learning approaches, future work could evaluate whether large language models can support annotation, abstract comprehension, or full-text interpretation. Because their outputs may vary with prompt design, model version, and generation parameters, such applications require standardized and prospectively validated protocols. Supervised fine-tuned classifiers may provide a more controlled basis for reproducible initial-screening evaluation, and the complementary use of both approaches warrants further study [60].

## 5. Conclusions

Within the retrieved and manually annotated corpus, biomedical domain-specific pretrained language models achieved higher mean WSS values and lower cross-fold variability than BERT-base at the complete-recall operating point, although these differences were not formally tested. Separately, the clinical review mapped frequently favorable but non-interchangeable discrimination estimates across endpoints, metric families, time horizons, units of analysis, and validation settings. The uniformly high analysis-domain risk of bias, incomplete uncertainty reporting, and limited independent validation indicate that the reported clinical performance remains insufficiently robust or transportable for routine use. Future research should prospectively validate the screening workflow and evaluate clinical prediction models using independent cohorts, calibration measures, and assessments of clinical utility.

## Figures and Tables

**Table 1:** Characteristics of the included studies.

Study	Year	Country/Region	Complication	Model	Primary Discrimination Estimate (95% CI)	Evaluation Cohort (*n*)	Validation Level
Bao et al. [19]	2022	China	RTLI	Radiomics-LR	ROC-AUC: 0.95	validation cohort (*n* = 80)	held-out internal validation
Bin et al. [20]	2022	China	RTLI	LR	Harrell’s C-index: 0.82 (0.71–0.92)	validation cohort (*n* = 59)	held-out internal validation
Guan et al. [21]	2024	China	TLI	NTCP-LR	ROC-AUC: 0.8702 (0.7577–0.9828)	validation set (*n* = 26)	held-out internal validation
Hong et al. [45]	2025	China	Radiation dermatitis	SVM	ROC-AUC: 0.797 (0.616–0.978)	test cohort (8:2 split of 161) (*n* = 32 (derived))	held-out internal validation (authors mislabel as external)
Hou et al. [18]	2024	China	CBRN	LR	ROC-AUC: 0.90	validation set (*n* = 59)	held-out internal validation
Hu et al. [29]	2024	China	SOM	H-MCF	ROC-AUC: 0.883	independent testing set (*n* = 40)	held-out internal validation (independent test set)
Huang et al. [27]	2023	China	Grade 4 lymphopenia	SVM	ROC-AUC: 0.93	test set (*n* = 40)	held-out internal validation
Huang et al. [46]	2023	China	RTLI	LR	ROC-AUC: 0.947	testing cohort (*n* = 39)	held-out internal validation
Lee et al. [38]	2014	Taiwan	Xerostomia	NTCP-LASSO	ROC-AUC: 0.87	NPC sub-cohort at the 3-month time point (*n* = NR)	bootstrap internal validation
Li et al. [30]	2020	China	SOM	LASSO-LR	ROC-AUC: 0.782	testing set (*n* = NR)	held-out internal validation
Li et al. [35]	2025	China	SOM	Dynamic BJM	Time-dependent AUC (1 month after completion of RT): 0.81 (0.68–0.87)	full cohort, bootstrap resampling (*n* = 294)	bootstrap internal validation
Li et al. [39]	2022	China	Xerostomia	RF	ROC-AUC: 0.915 (0.860–0.970)	testing set (*n* = 110)	held-out internal validation
Liang et al. [36]	2025	China	SOM	LR	ROC-AUC: 0.813 (0.752–0.875)	modelling cohort (January 2021–June 2022) (*n* = 192)	development/training only
Liu et al. [31]	2023	China	SOM	LR	ROC-AUC: 0.759 (0.691–0.827)	full cohort, apparent (*n* = 190)	development/training only
Nicol et al. [32]	2025	Multi-center	SOM	Multi-omic RF	ROC-AUC: 0.652 (0.529–0.767)	external validation cohort (*n* = 101)	external validation
Orlandi et al. [37]	2018	Italy	SOM	NTCP-LR/LASSO	ROC-AUC: 0.67	full cohort, bootstrap-corrected (*n* = 132)	bootstrap internal validation
OuYang et al. [22]	2025	China	TLI	NTCP-Poisson	ROC-AUC: 0.744	Data-B external validation dataset (*n* = 119)	external validation
Peng et al. [41]	2020	China	Hypothyroidism	LASSO-LR	ROC-AUC: 0.643 (0.590–0.695)	full cohort, apparent (*n* = 545)	development/training only
Qin et al. [47]	2023	China	Late xerostomia	Radiomics-SVM	ROC-AUC: 0.851 (0.697–0.999)	testing set (*n* = 25)	held-out internal validation
Quan et al. [42]	2024	China	Hypothyroidism	XGBoost	ROC-AUC: 0.842 (0.724–0.960)	test cohort (*n* = 44)	held-out internal validation
Ren et al. [23]	2024	China	TLI	3D CNN-LR	ROC-AUC: 0.843 (0.840–0.846)	outer CV folds over the whole cohort (*n* = 127)	cross-validation
Sun et al. [33]	2025	China	SOM	RF-OPM	ROC-AUC: 0.800	internal validation cohort (*n* = NR)	held-out internal validation
Teng et al. [48]	2021	China	Xerostomia (recovery)	NTCP-LASSO	ROC-AUC: 0.82 (0.70–0.95)	full cohort, 200 randomised LASSO test splits (*n* = 203)	bootstrap/repeated-split internal validation
Wang et al. [28]	2024	China	RTLI	Clinical-radiomics nomogram	ROC-AUC: 0.984	validation set (*n* = 69)	held-out internal validation
Wang et al. [49]	2024	China	Xerostomia	Radiomics-ΔLR	ROC-AUC: 0.702	test set (*n* = NR)	held-out internal validation
Wang et al. [50]	2025	China	RIHN	Fine-Gray	Harrell’s C-index: 0.691	testing set (*n* = 167)	held-out internal validation
Wang et al. [24]	2019	China	TLN	LASSO-LR	ROC-AUC: 0.685 (0.6048–0.765)	testing set (*n* = 493)	held-out internal validation (chronological split)
Wen et al. [25]	2021	China	TLI	Cox	Harrell’s C-index: 0.775 (0.751–0.799)	test set of temporal lobes (*n* = 4916)	held-out internal validation
Wongwattananard et al. [43]	2024	Thailand	Hypothyroidism	NTCP-MLR	ROC-AUC: 0.812	temporal validation cohort (*n* = 25)	temporal validation
Wungcharoen et al. [51]	2025	Thailand	Sensorineural hearing loss	NTCP-LR	ROC-AUC: 0.644	full cohort, 10-fold CV (*n* = 229)	cross-validation
Xiang et al. [52]	2025	China	Radiation dermatitis	DL-Dosiomics	ROC-AUC: 0.916 (0.866–0.967)	internal validation set (*n* = 72)	held-out internal validation
Yan et al. [53]	2021	China	ANCSA	NTCP-LASSO	ROC-AUC: 0.73 (0.64–0.82)	validation group (*n* = 183)	held-out internal validation
Yang et al. [40]	2021	China	Late xerostomia	LR	ROC-AUC: 0.811 (0.710–0.912)	full cohort, bootstrapped (*n* = 311)	bootstrap internal validation
Zeng et al. [34]	2025	China	SOM	MCF	ROC-AUC: 0.904	independent testing set (*n* = 86)	held-out internal validation (independent test set)
Zhang et al. [54]	2025	China	Xerostomia (recovery)	Cox	Time-dependent AUC (3 years (5-year value 0.81 also reported)): 0.773	validation set (*n* = 195)	held-out internal validation
Zhang et al. [26]	2026	China	RTLI	Ensemble (EN + RF + SVM)	ROC-AUC: 0.968	validation set (*n* = 48)	held-out internal validation
Zhong et al. [55]	2025	China	Late radiation injuries	LASSO-RSF	ROC-AUC: 0.8380 (0.806–0.858)	test group (*n* = 108)	held-out internal validation
Zhong et al. [44]	2025	China	Hypothyroidism	CoxBoost-RSF	Harrell’s C-index: 0.71	validation cohort (*n* = 141)	held-out internal validation

This table summarizes the author, publication year, country or region, predicted complication, model type, primary discrimination estimate, actual evaluation cohort size when reported, evaluation cohort, and audited validation level. The sample size refers to the cohort that generated the selected estimate rather than the total study population. NR indicates not reported. **Abbreviations:** ANCSA, anterior nasal cavity and sinus aperture stenosis; AUC, area under the curve; BJM, Bayesian joint model; CBRN, cystic brain radionecrosis; CI, confidence interval; CNN, convolutional neural network; Cox, Cox proportional hazards model; CoxBoost, Cox proportional hazards boosting model; DL, deep learning; EN, elastic net; Fine-Gray, Fine-Gray subdistribution hazard model; H-MCF, hierarchical multi-classifier fusion; LASSO, least absolute shrinkage and selection operator; LR, logistic regression; MCF, multi-classifier fusion; MLR, multivariable logistic regression; NTCP, normal tissue complication probability; OPM, optimal predictive model; RF, random forest; RIHN, radiation-induced hypoglossal neuropathy; RSF, random survival forest; RTLI, radiation-induced temporal lobe injury; SOM, severe oral mucositis; SVM, support vector machine; TLI, temporal lobe injury; TLN, temporal lobe necrosis; XGBoost, extreme gradient boosting; 3D, three-dimensional; ΔLR, delta logistic regression.

**Table 2:** Endpoint-specific clinical and predictor synthesis of the four major toxicity groups.

Endpoint	Primary OAR/Structure	Predictor Patterns Identified	Validation and Interpretation
Cerebral radiation injury or necrosis	Temporal lobe	Temporal-lobe dosimetric parameters, particularly small-volume high-dose metrics such as D_0.5cc_ [20,21,22,23,24,25]; age [18,19,20,21,24,25,26] and disease stage [18,19,23,24,25,26,27,28] were also commonly evaluated	Predominantly internally validated; endpoint definitions and analysis units varied
Severe oral mucositis	Oral cavity/oral mucosa	Oral-cavity or mucosal V_x_ metrics [29,30,31,32,33,34], BMI [30,32,35,36,37], and chemotherapy-related variables [31,32,33,35,37]	OAR delineation and grading systems varied; the reported direction of association for BMI was inconsistent
Xerostomia occurrence or severity	Primarily parotid glands	Parotid mean dose was the only specific predictor retained across multiple studies [38,39,40]	No external validation was identified; assessment time points and outcome definitions were heterogeneous
Hypothyroidism	Thyroid gland	Thyroid V_x_ metrics were recurrently retained or evaluated [41,42,43,44]; thyroid volume [41,44] and pretreatment TSH [43,44] were also evaluated	Evidence was based on four studies, with no cross-institutional external validation identified

This table provides an endpoint-specific clinical synthesis of the four major toxicity groups, including the primary organ at risk or anatomical structure, recurrent dosimetric and clinical predictor patterns, and major validation-related limitations. “Commonly evaluated” indicates that a variable was assessed in multiple studies and does not imply a consistent independent association or retention in the final model. “Recurrently retained” indicates that a variable was retained in the final models of more than one study but does not establish causality or a clinically applicable dose constraint. **Abbreviations:** BMI, body mass index; D_0.5cc_, dose received by the most exposed 0.5 cm^3^ of an organ; Gy, gray; OAR, organ at risk; TSH, thyroid-stimulating hormone; V_x_, volume or percentage of an organ receiving at least x Gy.

**Table 3:** Performance of pretrained language models for NLP-assisted screening.

	PubMedBERT	BioBERT	BERT-Base
ROC-AUC	0.995 ± 0.005	0.995 ± 0.004	0.983 ± 0.020
PR-AUC	0.647 ± 0.161	0.706 ± 0.200	0.664 ± 0.175
WSS@100%	97.50% ± 2.00%	97.80% ± 1.10%	89.70% ± 11.90%
WSS@85%	85.30% ± 0.90%	85.20% ± 1.10%	83.30% ± 3.50%

Values are presented as mean ± sample standard deviation across the five cross-validation folds. WSS@100% and WSS@85% indicate Work Saved over Sampling at the 100% and 85% recall operating points, respectively. **Abbreviations:** BERT, Bidirectional Encoder Representations from Transformers; BioBERT, Biomedical Bidirectional Encoder Representations from Transformers; CI, confidence interval; PR-AUC, area under the precision–recall curve; PubMedBERT, PubMed-pretrained Bidirectional Encoder Representations from Transformers; ROC-AUC, area under the receiver operating characteristic curve; SD, standard deviation; WSS, Work Saved over Sampling.

## Data Availability

The dataset, analysis code, and result files for the NLP-assisted title-and-abstract screening component of this study are available in Zenodo at https://zenodo.org/records/21768279 (accessed on 8 September 2026).

## Supplementary Materials

The following supporting information can be downloaded at: https://www.mdpi.com/article/10.3390/life16091566/s1. The supporting information includes Supplementary Appendix S1, Detailed search strategies for each database; Supplementary Table S1, PROBAST risk of bias assessment; Supplementary Table S2, Model performance at WSS@100% recall; Supplementary Table S3, Model performance at WSS@85% recall; Supplementary Table S4, Study-level discrimination metric, validation, and uncertainty audit; and Supplementary Table S5, Full-text excluded reports with reasons.
