# Supplementary material for: High-Recall Biomedical Language Models for Radiation Oncology Evidence Synthesis: An Integrated Systematic Review of Prediction Models for Radiotherapy-Induced Complications in Nasopharyngeal Carcinoma
Source: Life (Basel). 2026 Sep 18;16(9):1566. doi: 10.3390/life16091566 (PMC13608098; doi:10.3390/life16091566)
Supplement: Supplementary file 1 [file life-16-01566-s001.zip › life-4548039-supplementary.pdf]

## Supplementary Material

**Supplementary Appendix S1.** Detailed search strategies for each database.

| Database | Search strategy |
|---|---|
| **PubMed** | (("Nasopharyngeal Neoplasm"[tiab] OR "Neoplasm, Nasopharyngeal"[tiab] OR "Nasopharynx Neoplasms"[tiab] OR "Nasopharynx Neoplasm"[tiab] OR "Neoplasm, Nasopharynx"[tiab] OR "Neoplasms, Nasopharynx"[tiab] OR "Neoplasms, Nasopharyngeal"[tiab] OR "Cancer of Nasopharynx"[tiab] OR "Nasopharynx Cancers"[tiab] OR "Cancer of the Nasopharynx"[tiab] OR "Nasopharyngeal Cancer"[tiab] OR "Cancer, Nasopharyngeal"[tiab] OR "Cancers, Nasopharyngeal"[tiab] OR "Nasopharyngeal Cancers"[tiab] OR "Nasopharynx Cancer"[tiab] OR "Cancer, Nasopharynx"[tiab] OR "Cancers, Nasopharynx"[tiab] OR "Nasopharyngeal Carcinoma"[tiab] OR "NPC"[tiab])<br>AND ("RT"[tiab] OR "Radiotherapy"[tiab] OR "Radiation Therapy"[tiab] OR "Radiation Treatment"[tiab] OR "Radiotherapy, Intensity-Modulated"[tiab] OR "Intensity-Modulated Radiotherapy"[tiab] OR "IMRT"[tiab] OR "Volumetric-Modulated Arc Therapy"[tiab] OR "VMAT"[tiab] OR "Helical Tomotherapy"[tiab] OR "Tomotherapy"[tiab])<br>AND ("machine learning"[tiab] OR "Learning, Machine"[tiab] OR "deep learning"[tiab] OR "Learning, Deep"[tiab] OR "transfer learning"[tiab] OR "Learning, Transfer"[tiab] OR "hierarchical learning"[tiab] OR "Learning, Hierarchical"[tiab] OR "ML"[tiab] OR "DL"[tiab] OR "model"[tiab] OR "predict"[tiab] OR "classifier"[tiab])<br>AND ("NTCP"[tiab] OR "Normal Tissue Complication Probability"[tiab] OR "xerostomia*"[tiab] OR "hyposalivation*"[tiab] OR "oral mucositis"[tiab] OR "stomatitis"[tiab] OR "radiodermatitis"[tiab] OR "radiation-induced dermatitis"[tiab] OR "temporal lobe injury"[tiab] OR "temporal lobe necrosis"[tiab] OR "radiation-induced temporal lobe injury"[tiab] OR "hypothyroidism"[tiab] OR "lymphopenia"[tiab] OR "radiation-induced lymphopenia"[tiab] OR "hearing loss"[tiab] OR "middle ear effusion"[tiab] OR "brain radionecrosis"[tiab] OR "radionecrosis"[tiab] OR "hypoglossal neuropathy"[tiab] OR "nasal cavity stenosis"[tiab] OR "nasal cavity atresia"[tiab] OR "late radiation injury"[tiab] OR "late toxicity"[tiab] OR "radiation toxicity"[tiab])) |
| **Web of Science** | TS=(("Nasopharyngeal Carcinoma*" OR "NPC" OR "Nasopharyngeal Neoplasm*" OR "Nasopharynx Cancer*")<br>AND ("RT" OR "Radiotherapy" OR "Radiation Therapy" OR "Radiation Treatment" OR "IMRT" OR "VMAT" OR "Tomotherapy")<br>AND ("machine learning" OR "deep learning" OR "ML" OR "DL" OR "model*" OR "predict*" OR "classifier*" OR "transfer learning" OR "hierarchical learning") |

| | |
|---|---|
| | AND ("NTCP" OR "Normal Tissue Complication Probability" OR "xerostomia*" OR "hyposalivation*" OR "oral mucositis" OR "stomatitis" OR "radiodermatitis" OR "radiation-induced dermatitis" OR "temporal lobe injury" OR "temporal lobe necrosis" OR "radiation-induced temporal lobe injury" OR "hypothyroidism" OR "lymphopenia" OR "radiation-induced lymphopenia" OR "hearing loss" OR "middle ear effusion" OR "brain radionecrosis" OR "radionecrosis" OR "hypoglossal neuropathy" OR "nasal cavity stenosis" OR "nasal cavity atresia" OR "late radiation injury" OR "late toxicity" OR "radiation toxicity")) |
| **Scopus** | TITLE-ABS-KEY (<br>("Nasopharyngeal Carcinoma*" OR "NPC" OR "Nasopharyngeal Neoplasm*" OR "Nasopharynx Cancer*")<br>AND ("RT" OR "Radiotherapy" OR "Radiation Therapy" OR "Radiation Treatment" OR "IMRT" OR "VMAT" OR "Tomotherapy")<br>AND ("machine learning" OR "deep learning" OR "ML" OR "DL" OR "model*" OR "predict*" OR "classifier*" OR "transfer learning" OR "hierarchical learning")<br>AND ("NTCP" OR "Normal Tissue Complication Probability" OR "xerostomia*" OR "hyposalivation*" OR "oral mucositis" OR "stomatitis" OR "radiodermatitis" OR "radiation-induced dermatitis" OR "temporal lobe injury" OR "temporal lobe necrosis" OR "radiation-induced temporal lobe injury" OR "hypothyroidism" OR "lymphopenia" OR "radiation-induced lymphopenia" OR "hearing loss" OR "middle ear effusion" OR "brain radionecrosis" OR "radionecrosis" OR "hypoglossal neuropathy" OR "nasal cavity stenosis" OR "nasal cavity atresia" OR "late radiation injury" OR "late toxicity" OR "radiation toxicity")<br>) |
| **Cochrane Library** | ((Nasopharyngeal NEXT Carcinoma*) OR NPC OR (Nasopharyngeal NEXT Neoplasm*) OR (Nasopharynx NEXT Cancer*)):ti,ab,kw AND (RT OR Radiotherapy OR (Radiation NEXT Therapy) OR (Radiation NEXT Treatment) OR IMRT OR VMAT OR Tomotherapy)):ti,ab,kw AND ((machine NEXT learning) OR (deep NEXT learning) OR ML OR DL OR model* OR predict* OR classifier* OR (transfer NEXT learning) OR (hierarchical NEXT learning)):ti,ab,kw AND (NTCP OR (Normal NEXT Tissue NEXT Complication NEXT Probability) OR xerostomia* OR hyposalivation* OR (oral NEXT mucositis) OR stomatitis OR radiodermatitis OR (radiation NEXT induced NEXT dermatitis) OR (temporal NEXT lobe NEXT injury) OR (temporal NEXT lobe NEXT necrosis) OR (radiation NEXT induced NEXT temporal NEXT lobe NEXT injury) OR hypothyroidism OR lymphopenia OR (radiation NEXT induced NEXT lymphopenia) OR (hearing NEXT loss) OR (middle NEXT ear NEXT effusion) OR (brain NEXT radionecrosis) OR radionecrosis OR (hypoglossal NEXT neuropathy) OR (nasal NEXT cavity NEXT stenosis) OR (nasal NEXT cavity NEXT atresia) OR (late NEXT radiation NEXT injury) OR (late NEXT toxicity) OR (radiation NEXT toxicity)):ti,ab,kw |

**Supplementary Table S1.** PROBAST risk of bias assessment.

| Author (Year) | Risk of Bias | | | | Applicability | | | Overall | |
|---|---|---|---|---|---|---|---|---|---|
| | 1. Participants | 2. Predictors | 3. Outcome | 4. Analysis | 1. Participants | 2. Predictors | 3. Outcome | Risk of Bias | Applicability |
| Bao et al. (2022) | + | ? | - | - | - | + | + | - | - |
| Bin et al. (2022) | + | + | + | - | + | + | + | - | + |
| Guan et al. (2024) | + | + | + | - | + | + | + | - | + |
| Hong et al. (2025) | + | + | + | - | + | + | + | - | + |
| Hou et al. (2024) | - | + | + | - | + | + | + | - | + |
| Hu et al. (2024) | + | + | + | - | + | + | + | - | + |
| Huang et al. (2023) | + | + | + | - | + | + | + | - | + |
| Huang et al. (2023) | - | + | + | - | - | + | + | - | - |
| Lee et al. (2014) | + | + | + | - | + | + | + | - | + |
| Li et al. (2020) | + | + | + | - | + | + | + | - | + |
| Li et al. (2025) | + | + | + | - | + | + | + | - | + |

| | | | | | | | | | |
|---|---|---|---|---|---|---|---|---|---|
| Li et al. (2022) | + | + | + | - | + | + | + | - | + |
| Liang et al. (2025) | + | + | + | - | + | + | + | - | + |
| Liu et al. (2023) | + | + | + | - | + | + | + | - | + |
| Nicol et al. (2025) | + | + | + | - | + | + | + | - | + |
| Orlandi et al. (2018) | + | + | + | - | + | + | + | - | + |
| OuYang et al. (2025) | + | + | ? | - | + | + | + | - | + |
| Peng et al. (2020) | - | + | + | - | + | + | + | - | + |
| Qin et al. (2023) | + | + | + | - | + | + | + | - | + |
| Quan et al. (2024) | + | + | + | - | + | + | + | - | + |
| Ren et al. (2024) | + | + | + | - | + | + | + | - | + |
| Sun et al. (2025) | + | + | + | - | + | + | + | - | + |
| Teng et al. (2021) | + | + | + | - | + | + | + | - | + |
| Wang et al. (2024) | - | + | + | - | - | + | + | - | - |
| Wang et al. (2025) | + | + | + | - | + | + | + | - | + |
| Wang et al. (2024) | - | + | + | - | - | + | + | - | - |

| | | | | | | | | | |
|---|---|---|---|---|---|---|---|---|---|
| Wang et al. (2019) | + | + | + | - | + | + | + | - | + |
| Wen et al. (2021) | + | + | + | - | + | + | + | - | + |
| Wongwattananard et al. (2024) | + | + | + | - | + | + | + | - | + |
| Wungcharoen et al. (2025) | - | + | + | - | + | + | + | - | + |
| Xiang et al. (2025) | + | + | + | - | + | + | + | - | + |
| Yan et al. (2021) | + | + | + | - | + | + | + | - | + |
| Yang et al. (2021) | + | + | + | - | + | + | + | - | + |
| Zeng et al. (2025) | + | + | + | - | + | + | + | - | + |
| Zhang et al. (2025) | + | + | + | - | + | + | + | - | + |
| Zhang et al. (2026) | + | + | + | - | + | + | + | - | + |
| Zhong et al. (2025) | + | + | + | - | + | + | + | - | + |
| Zhong et al. (2025) | + | + | + | - | + | + | + | - | + |

This table summarizes the risk of bias and applicability assessment of the included prediction model studies across PROBAST domains. **Abbreviations:** +, low risk / low concern; −, high risk / high concern; ?, unclear; PROBAST, Prediction Model Risk of Bias Assessment Tool.

Summary of analysis-domain judgments: High-risk judgments most frequently reflected predictor selection based on univariable analyses (20 studies), absence of calibration assessment (14 studies), and clearly inadequate sample sizes or event counts relative to model complexity (12 studies); a further 13 studies raised possible sample-size concerns. Additional concerns included incomplete control of overfitting or optimism and inadequate handling or reporting of censoring,

clustered observations, missing data, and the independence of preprocessing, feature selection, and validation. Confirmed evidence of data leakage was limited to one study; inadequate reporting was not interpreted as proof that data leakage had occurred.

**Supplementary Table S2.** Model performance at WSS@100% recall.

| | PubMedBERT | BioBERT | BERT-base |
|---|---|---|---|
| Recall | 1.000 ± 0.000 | 1.000 ± 0.000 | 1.000 ± 0.000 |
| Precision | 0.406 ± 0.162 | 0.442 ± 0.223 | 0.295 ± 0.260 |
| Specificity | 0.982 ± 0.020 | 0.986 ± 0.010 | 0.904 ± 0.119 |
| NPV | 1.000 ± 0.000 | 1.000 ± 0.000 | 1.000 ± 0.000 |
| WSS | 97.50% ± 2.00% | 97.80% ± 1.10% | 89.70% ± 11.90% |
| Burden | 2.50% ± 2.00% | 2.20% ± 1.10% | 10.30% ± 11.90% |
| n_Screened | 24.8 ± 19.8 | 21.2 ± 10.4 | 101.2 ± 116.8 |

Values are presented as mean ± sample standard deviation across the five cross-validation folds. This table reports recall, precision, specificity, negative predictive value, WSS, screening burden, and number of screened records for each pretrained language model at the 100% recall operating point.
**Abbreviations**: BERT, Bidirectional Encoder Representations from Transformers; BioBERT, Biomedical Bidirectional Encoder Representations from Transformers; NPV, negative predictive value; n_Screened, number of records screened; PubMedBERT, PubMed-pretrained Bidirectional Encoder Representations from Transformers; WSS, Work Saved over Sampling.

**Supplementary Table S3.** Model performance at WSS@85% recall.

| | PubMedBERT | BioBERT | BERT-base |
|---|---|---|---|
| Recall | 0.868 ± 0.010 | 0.868 ± 0.010 | 0.868 ± 0.010 |

| | | | |
|---|---|---|---|
| Precision | 0.575 ± 0.239 | 0.520 ± 0.247 | 0.424 ± 0.305 |
| Specificity | 0.992 ± 0.010 | 0.991 ± 0.009 | 0.972 ± 0.040 |
| NPV | 0.999 ± 0.000 | 0.999 ± 0.000 | 0.999 ± 0.000 |
| WSS (%) | 85.30 ± 0.90 | 85.20 ± 1.10 | 83.30 ± 3.50 |
| Burden (%) | 1.50 ± 1.00 | 1.60 ± 0.90 | 3.50 ± 4.00 |
| n_Screened | 14.6 ± 10.0 | 15.8 ± 8.9 | 34.4 ± 38.9 |

Values are presented as mean ± sample standard deviation across the five cross-validation folds. This table reports recall, precision, specificity, negative predictive value, WSS, screening burden, and number of screened records for each pretrained language model at the 85% recall operating point. **Abbreviations:** BERT, Bidirectional Encoder Representations from Transformers; BioBERT, Biomedical Bidirectional Encoder Representations from Transformers; NPV, negative predictive value; n_Screened, number of records screened; PubMedBERT, PubMed-pretrained Bidirectional Encoder Representations from Transformers; WSS, Work Saved over Sampling. The nominal 85% recall operating point represents the minimum ranking position that achieved at least 85% recall; because the number of relevant records per fold was discrete, the mean recall actually achieved was 0.868 ± 0.010.

**Supplementary Table S4. Study-level discrimination metric, validation, and uncertainty audit.**

| Study | Endpoint | Metric | Time horizon | Estimate (95% CI) | Evaluation n | Events/non-events | Validation | Grade | Source |
|---|---|---|---|---|---|---|---|---|---|
| Bao 2022 | cerebral radiation injury | conventional binary AUC | not applicable (binary, any RTLI during follow-up) | 0.95 | 80 | 32 / 48 | held-out internal validation | B | p1 Abstract; p6 performance table (sens 0.81=26/32, spec 0.96=46/48) |
| Bin 2022 | cerebral radiation injury | conventional binary AUC (equivalent) | not applicable | 0.82 (0.71-0.92) | 59 | not reported for the split / not reported for the split | held-out internal validation | B | p1 Abstract; p3 Methods (Harrell's concordance index); p5 Results |
| Guan 2024 | cerebral radiation injury | conventional binary AUC | not applicable | 0.8702 (0.7577-0.9828) | 26 | not reported for the split / not reported for the split | held-out internal validation | B | Abstract and Results/ROC table |
| Hong 2025 | radiation dermatitis | conventional binary AUC | not applicable | 0.797 (0.616-0.978) | 32 (derived) | 11 / 21 | held-out internal validation (authors mislabel as external) | E | p4 (11 patients, 34.38%); p7-p8 and p11 AUC values |
| Hou 2024 | cerebral brain radionecrosis | conventional binary AUC | not applicable | 0.90 | 59 | not reported for the split / not reported for the split | held-out internal validation | B | p1 Abstract; p6 Results; p8 AUC (95%CI) table |

| | | | | | | | | | |
|---|---|---|---|---|---|---|---|---|---|
| Hu 2024 | severe oral mucositis | conventional binary AUC | not applicable | 0.883 | 40 | 15 (derived) / 25 (derived) | held-out internal validation (independent test set) | C | p1 Abstract (AUC 0.883, ACC 0.850, SEN 0.933, SPE 0.800); p2/p3 'Independent testing set'; p5 evaluation-metric section |
| Huang 2023 | grade 4 lymphopenia | conventional binary AUC | not applicable | 0.93 | 40 | not reported for the split / not reported for the split | held-out internal validation | D | p5 Results; p7 Mean AUC (95% CI) table |
| Huang 2023 | cerebral radiation injury | conventional binary AUC | not applicable | 0.947 | 39 | not reported for the split / not reported for the split | held-out internal validation | B | p5 Results (combined 0.962 [0.9306-0.9939] training; 0.947 [0.8841-...] testing) |
| Lee 2014 | xerostomia occurrence or severity | conventional binary AUC | 3 months after RT (a questionnaire time point, not a time-to-event horizon) | 0.87 | NR | not reported / not reported | bootstrap internal validation | E | p4 Results ('For the NPC model, the AUC was 0.87 and 0.96 for the time...'); Tables 4-5 |
| Li 2020 | severe oral | conventional | not | 0.782 | NR | not reported | held-out internal | E | p1 Abstract; p3-p4 |

| | | | | | | | | | |
|---|---|---|---|---|---|---|---|---|---|
| | mucositis | binary AUC | applicable | | | / not reported | validation | | Results; p7 table footnote |
| Li 2022 | xerostomia occurrence or severity | conventional binary AUC | not applicable (assessment window not reported) | 0.915 (0.860-0.970) | 110 | not reported for the split / not reported for the split | held-out internal validation | B | p1 Abstract; p7 Results and AUC (95%CI) table; p10 table |
| Li 2025 | severe oral mucositis | time dependent AUC | 1 month after completion of RT (also reported at 2 months = 0.82 and 3 months = 0.81) | 0.81 (0.68-0.87) | 294 | not reported per time point / not reported per time point | bootstrap internal validation | B | p1 Abstract; p2 Methods (100 bootstrap iterations, time-dependent AUC); p4 Results (0.81 [95%CI 0.68-0.87], 0.82, 0.81) |
| Liang 2025 | severe oral mucositis | conventional binary AUC | not applicable | 0.813 (0.752-0.875) | 192 | not cleanly reported (p2 and p4 give contradictory SRIOM/non-SRIOM labels, 112 vs 80 swapped) / see above | development/training only | B | p1 Abstract; p4 Results (Logit(P)>0.474, AUC 0.813 [95% CI 0.752-0.875]) |

| | | | | | | | | | |
|---|---|---|---|---|---|---|---|---|---|
| Liu 2023 | severe oral mucositis | conventional binary AUC | not applicable | 0.759 (0.691-0.827) | 190 | not reported / not reported | development/training only | B | p1 Abstract; p3 (no external verification); p7 Results |
| Nicol 2025 | severe oral mucositis | conventional binary AUC | not applicable | 0.652 (0.529-0.767) | 101 | not reported for the external cohort / not reported for the external cohort | external validation | B | p1 Abstract (0.67 [0.61,0.73] internal; 0.65 [0.53,0.77] external); p6 Results |
| Orlandi 2018 | severe oral mucositis | conventional binary AUC | not applicable | 0.67 | 132 | not reported / not reported | bootstrap internal validation | D | p3 Results (apparent AUC 0.69; bootstrap-corrected 0.67); p4 (second endpoint 0.61/0.60) |
| OuYang 2025 | cerebral radiation injury | conventional binary AUC | not applicable | 0.744 | 119 | not reported / not reported | external validation | D | p9 Methods (AUC and coefficient of determination); results tables of the 45 combinations |
| Peng 2020 | hypothyroidism | conventional binary AUC | 2 years (landmark; achieved by excluding | 0.643 (0.590-0.695) | 545 | not reported in extractable text / not reported in | development/training only | B | p2-p3 and p6 (censoring rule); p5 AUC (95% CI) table |

| | | | | | | | | | |
|---|---|---|---|---|---|---|---|---|---|
| | | | those censored earlier, not by time-to-event modelling) | | | extractable text | | | |
| Qin 2023 | xerostomia occurrence or severity | conventional binary AUC | 'late' (exact assessment time not specified) | 0.851 (0.697-0.999) | 25 | not reported / not reported | held-out internal validation | B | p1 Abstract; p4-p5 AUC (95% CI) tables |
| Quan 2024 | hypothyroidism | conventional binary AUC | not applicable | 0.842 (0.724-0.960) | 44 | not reported for the split / not reported for the split | held-out internal validation | B | p5-p7 Results (training 1.000 [95% CI ...]; test cohort values) |
| Ren 2024 | cerebral radiation injury | conventional binary AUC | not applicable | 0.843 (0.840-0.846) | 127 | not reported / not reported | cross-validation | E | p1 Abstract; p3 Methods (nested CV, 'AUC with a 95%...') |
| Sun 2025 | severe oral mucositis | conventional binary AUC | not applicable | 0.800 | NR | not reported / not reported | held-out internal validation | E | p1 Abstract (validation 0.800; test cohort 0.670); p2 (test cohort n=88); p5 Results |
| Teng 2021 | xerostomia recovery | conventional binary AUC | 2 years (recovery endpoint) | 0.82 (0.70-0.95) | 203 | not reported / not reported | bootstrap/repeated-split internal validation | B (with an estimand | p8 (AUC index for R50-1year and R80-2years) |

| | | | | | | | | | |
|---|---|---|---|---|---|---|---|---|---|
| | | | | | | | | caveat) | |
| Wang 2019 | cerebral radiation injury | conventional binary AUC | not applicable | 0.685 (0.6048-0.765) | 493 | not reported for the split (38 TLN cases overall, 32/38 grade 1) / not reported for the split | held-out internal validation (chronological split) | B | p1 Abstract (C-index 0.685 [95% CI 0.6048-0.765]); p3 Results ('AUC for testing set is 0.6849 (95% CI: 0.6048-0.765)') |
| Wang 2024 | cerebral radiation injury | conventional binary AUC | not applicable | 0.984 | 69 | not reported for the split / not reported for the split | held-out internal validation | D | p2 Abstract (AUC 0.984 in the validation set); p3 (training n=169, validation n=69); p6 Results |
| Wang 2024 | xerostomia occurrence or severity | conventional binary AUC | within 1 week after CCRT (ACUTE; not comparable with the late xerostomia studies) | 0.702 | NR | not reported for the split / not reported for the split | held-out internal validation | E | p4 Methods (7:3 split); p5 and p7 Table 2 (training and test set verification results) |
| Wang 2025 | radiation-induced | C index time to event | nomogram targets 8-year | 0.691 | 167 | not reported for the split / | held-out internal validation | D | p1 Abstract; p2 Methods (Harrell's C- |

| | | | | | | | | | |
|---|---|---|---|---|---|---|---|---|---|
| | hypoglossal neuropathy | | cumulative incidence; the C-index itself is horizon-free | | | not reported for the split | | | index; 8-year cumulative incidence nomogram); p3 (256/167 split); p5-p6 Results |
| Wen 2021 | cerebral radiation injury | C index time to event | horizon-free (C-index over the whole follow-up; median follow-up 66.8 months) | 0.775 (0.751-0.799) | 4916 | 387 / 4529 | held-out internal validation | B | p1 Abstract; p3 Methods (Cox, C-index, censoring rule); p4 col.1 Results (test set 0.775 [95% CI 0.751-0.799]); p4 (387/4916 = 7.9% injured TLs) |
| Wen 2021 | cerebral radiation injury | time dependent AUC | 5 years | 0.818 | 4916 | 387 total over follow-up; the number of events observed by exactly 5 years is NOT reported / not derivable at the 5-year | held-out internal validation | D | p4 col.1 Results ('better 5-year AUC (train set: 0.832 vs 0.783; test set: 0.818 vs 0.771; both P<0.001; Supplementary Tab. 4)') |

| | | | | | | | | | |
|---|---|---|---|---|---|---|---|---|---|
| | | | | | | horizon (subject to censoring) | | | |
| Wongwattananard 2024 | hypothyroidism | conventional binary AUC | not applicable | 0.812 | 25 | not reported / not reported | temporal validation | D | p1 Abstract (0.749 internal n=200; 0.812 external n=25); p3 Results (development AUROC 0.749 [95% CI 0.677-0.821]; 10-fold CV mean 0.764 [95% CI 0.634-0.795]; external n=25 AUROC 0.812, no CI) |
| Wungcharoen 2025 | sensorineural hearing loss | conventional binary AUC | not applicable | 0.644 | 229 | not reported / not reported | cross-validation | D | p5 Results (AUC 0.644 with precision ...) |
| Xiang 2025 | radiation dermatitis | conventional binary AUC | not applicable | 0.916 (0.866-0.967) | 72 | not reported for the split / not reported for the split | held-out internal validation | B | p2 (set sizes); p4 AUC (95%CI) table; p5-p6 Results |
| Yan 2021 | acquired nasal cavity stenosis and atresia | conventional binary AUC | not applicable | 0.73 (0.64-0.82) | 183 | not reported for the split / not reported | held-out internal validation | B | p1 Abstract (training 0.79 [95%CI 0.73-0.84]); p5 Results |

| | | | | | | | | | |
|---|---|---|---|---|---|---|---|---|---|
| | | | | | | for the split | | | (validation 0.73 [95%CI 0.64-0.82]) |
| Yang 2021 | xerostomia occurrence or severity | conventional binary AUC | 2 years (landmark) | 0.811 (0.710-0.912) | 311 | not reported / not reported | bootstrap internal validation | B | p2 Methods (censoring/landmark rules); Results AUC with 95% CI |
| Zeng 2025 | severe oral mucositis | conventional binary AUC | not applicable | 0.904 | 86 | 34 (derived) / 52 (derived) | held-out internal validation (independent test set) | C | p1 Abstract and p3 Results (AUC 0.904, ACC 0.849, SEN 0.853, SPE 0.846, independent testing set, Table 2/Fig. 2); p2 Methods (training/validation + independent testing split) |
| Zhang 2025 | xerostomia recovery | time dependent AUC | 3 years (5-year value 0.81 also reported) | 0.773 | 195 | not reported at the horizon / not reported at the horizon | held-out internal validation | D | p1 Abstract; p3-p4 Results (3-year/5-year AUC 0.77/0.806 training; 0.773/0.81 validation); p6 Fig. 3 |
| Zhang 2026 | cerebral radiation injury | conventional binary AUC | not applicable | 0.968 | 48 | not reported for the split / not reported | held-out internal validation | B | p6 AUC (95 %CI) tables |

| | | | | | | | | | |
|---|---|---|---|---|---|---|---|---|---|
| | | | | | | for the split | | | |
| Zhong 2025 | composite late radiation injuries | conventional binary AUC | not applicable | 0.8380 (0.806-0.858) | 108 | not reported for the split / not reported for the split | held-out internal validation | B (flagged) | p1 Abstract; p6 Methods (pROC/ROCR); p8 Results (training 0.9126 [95% CI 0.889-0.924]; test 0.8380 [95% CI 0.806-0.858]) |
| Zhong 2025 | hypothyroidism | Harrell's C-index (primary) | not applicable | 0.71 | 141 | not reported / not reported | held-out internal validation | D | p1 Abstract (C-index 0.91 training / 0.71 validation); p3 (328/141 split) |

Notes: Grade A, directly reported standard error or variance; Grade B, reported 95% confidence interval; Grade C, conventional binary AUC with complete event and non-event counts for the evaluation cohort; Grade D, point estimate only; Grade E, unclear metric definition, time horizon, evaluation cohort, or numerical provenance. Confidence intervals were transcribed as reported in the original publications and were not recalculated; standard errors were not reconstructed from AUC and sample size. Metrics and horizons are retained as reported and were not statistically pooled. NR, not reported.

**Supplementary Table S5.** Full-text excluded reports with reasons

| No. | First author / Year | Title | Identifier | Reason for exclusion |
|---|---|---|---|---|
| 1 | Zhou et al., 2024 | Establishment of a predictive model for severe acute radiotherapy adverse reactions in nasopharyngeal carcinoma patients based on Olink proteomics | 10.3760/cma.j.cn115355-20240124-00051 | Full text could not be obtained |
| 2 | Zhou et al., 2017 | Apparent diffusion coefficient histogram analysis can evaluate radiation-induced parotid damage and | 10.18632/oncotarget.19602 | Did not report AUC as a measure of discriminative |

| | | | | |
|---|---|---|---|---|
| | | predict late xerostomia degree in nasopharyngeal carcinoma | | performance |
| 3 | Zhou et al., 2022 | Integrated radiomics, dose-volume histogram criteria and clinical features for early prediction of saliva amount reduction after radiotherapy in nasopharyngeal cancer patients | 10.1007/s12672-022-00606-x | Did not report AUC as a measure of discriminative performance |
| 4 | Zhang et al., 2025 | Oral dose-volume parameters as independent predictors of severe radiation-induced mucositis in nasopharyngeal carcinoma patients: a retrospective study | 10.62347/swvq2652 | Did not report AUC as a measure of discriminative performance |
| 5 | Zhang et al., 2020 | Machine-learning based MRI radiomics models for early detection of radiation-induced brain injury in nasopharyngeal carcinoma | 10.1186/s12885-020-06957-4 | Did not report AUC as a measure of discriminative performance |
| 6 | Zeng et al., 2025 | Normal Tissue Complication Probability Model for Radiation-induced Temporal Lobe Injury after Intensity-modulated Radiation Therapy for Nasopharyngeal Carcinoma (Erratum, vol 276, 2015) | 10.1148/radiol.259014 | Full text could not be obtained |
| 7 | Zeng et al., 2015 | Normal Tissue Complication Probability Model for Radiation-induced Temporal Lobe Injury after Intensity-modulated Radiation Therapy for Nasopharyngeal Carcinoma | 10.1148/radiol.14141721 | Did not report AUC as a measure of discriminative performance |
| 8 | Zeng, 2018 | Comparing different normal tissue complication probability (NTCP) models of radiation-induced temporal lobe injury after intensity-modulated radiation therapy for nasopharyngeal carcinoma | WOS:000458082200369 | Conference abstract, not original research |
| 9 | Yan et al., 2025 | Predictive Modeling of Radiation-Induced Dermatitis | WOS:001596131800121 | Conference abstract, not |

| | | | | |
|---|---|---|---|---|
| | | in Nasopharyngeal Carcinoma Patients Undergoing TomoTherapy Using Machine Learning with Multimodal Data Integration | | original research |
| 10 | Wang et al., 2021 | Development of a normal tissue complication probability (NTCP) model using an artificial neural network for radiation-induced necrosis after carbon ion re-irradiation in locally recurrent carcinoma | 10.21037/atm-20-7805 | Use of non-modern photon IMRT technique (particle/carbon ion therapy) |
| 11 | Wang et al., 2023 | Delta radiomics based on fat suppression contrast enhanced T1WI for predicting xerostomia grade of nasopharyngeal carcinoma patients after radiotherapy | 10.13929/j.issn.1003-3289.2023.05.006 | Full text could not be obtained |
| 12 | Song et al., 2024 | Prediction of severe radiation-induced oral mucositis in locally advanced nasopharyngeal carcinoma using the combined systemic immune-inflammatory index and prognostic nutritional index | 10.1007/s00405-024-08536-w | Did not report AUC as a measure of discriminative performance |
| 13 | Qin et al., 2022 | Prediction of acute radiation-induced xerostomia grading of nasopharyngeal carcinoma based on multi-sequence MRI radiomics of the parotid gland | 10.16073/j.cnki.cjcpt.2022.22.05 | Full text could not be obtained |
| 14 | Qian et al., 2020 | Differences in lower cranial nerve complications predicted by the NTCP model between RTOG and reduced-volume IMRT planning in radiotherapy for nasopharyngeal carcinoma | 10.21037/tcr.2019.12.75 | Topic mismatch - comparison of treatment-planning techniques, not model development/validation |
| 15 | Luo et al., 2005 | Analysis of oral mucositis risk factors during radiotherapy for nasopharyngeal carcinoma patients and establishment of a discriminant model | PMID: 16004814 | Full text could not be obtained |
| 16 | Liu et al., 2019 | Early prediction of acute xerostomia during radiation therapy for nasopharyngeal cancer based on delta | 10.21037/qims.2019.07.08 | Did not report AUC as a measure of discriminative |

| | | | | |
|---|---|---|---|---|
| | | radiomics from CT images | | performance |
| 17 | Liu et al., 2025 | Dosiomics model for predicting radiation-induced temporal lobe injury in nasopharyngeal carcinoma after intensity-modulated radiotherapy | 10.3760/cma.j.cn113030-20240710-00267 | Full text could not be obtained |
| 18 | Li et al., 2017 | Oral Mucosa Dose Parameters Predicting Grade>=3 Acute Toxicity in Locally Advanced Nasopharyngeal Carcinoma Patients Treated With Concurrent IMRT and Chemotherapy | 10.1016/j.tranon.2017.06.011 | Did not report AUC as a measure of discriminative performance |
| 19 | Jian-Jun et al., 2016 | Delineation of Lower Cranial Nerves and NTCP Prediction From RTOG-Based and China-Based Target Definition Methods of IMRT Planning in Radiation Therapy of Nasopharyngeal Carcinoma | 10.1016/j.ijrobp.2016.06.1599 | Conference abstract, not original research |
| 20 | Feng et al., 2022 | Early Prediction Model of Radiation-Induced Xerostomia Based on Radiomics during Radiotherapy for Nasopharyngeal Carcinoma | WOS:000892639302123 | Conference abstract, not original research |
| 21 | Dong et al., 2023 | Multimodal Data Integration to Predict Severe Acute Oral Mucositis of Nasopharyngeal Carcinoma Patients Following Radiation Therapy | 10.3390/cancers15072032 | Full text could not be obtained |
